# 

**Published:** 1828-01-01

**Authors:** 


### THE MEDICO-CHIRURGICAL REVIEW,

AND

### JOURNAL

OF

### PRACTICAL MEDICINE.

*(NEW SERIES.)*

VOLUME EIGHT,

[1st of JANUARY to 30th of MARCH,]

1828.

VOL. I. OF FASCICULAR SERIES.

EDITED BY JAMES JOHNSON, M.D.

Surgeon Gen'l's Office
LIBRARY
57443
Washington, D.C.

LONDON;
PUBLISHED BY S. HIGHLEY, 174, FLEET STREET,
AND WEBB STREET, ST. THOMAS'S HOSPITAL,
And all other Booksellers.

W1
ME 75 T

G. HAYDEN.
Little College Street, Westminster.

### BIBLIOGRAPHICAL RECORD;

OR,

*Works received for Review from the 15th of September to the 15th of December, 1827.*

1. Manual of Pathology; containing the Symptoms, Diagnosis, and Morbid Characters of Diseases; together with an Exposition of the different Methods of Examination, applicable to Affections of the Head, Chest, and Abdomen. By L. Martinet, D. M. P. Translated, with Notes and Additions, by Jones Quain, A.B. Demonstrator of Anatomy at the Medical School, Aldersgate Street. Second Edition, revised, with additional Notes. 18mo, pp. 297. London, Simpkin and Marshal, 1827.

☞ *We are glad to see that the favourable opinion which we expressed of this Work has been confirmed by the rapid sale of the first edition. This second edition is considerably improved, and is still more entitled to public patronage.*

2. Rambling Notes and Reflections, suggested during a Visit to Paris in the Winter of 1826-1827. By Sir Arthur Brooke Faulkner. 8vo, pp. 348. London, Longman, 1827.

☞ *See the present No. of this Journal.*

3. An Introduction to the Comparative Anatomy of Animals; compiled with constant Reference to Physiology, and elucidated by Twenty Copper-plates. By C. G. Carus, Med. et Phil. Doct. Professor of Midwifery to the Medico-Chirurgical Academy at Dresden, &c Translated from the German, by R. T. Gore, M. R. C. S. In Two Volumes, 8vo, pp. 371—400, with one Vol. of Plates, in 4to, pp. 50. Longman, London, 1827.

☞ *This Work is worth its weight in gold. The diagrams and plans are so numerous and well managed, as to render the study of comparative anatomy throughout the whole range of animated nature, a matter of complete facility. It is a great acquisition to the zoological science and literature of this country.*

4. A Manual of Comparative Anatomy; translated from the German of J. F. Blumenbach, with additional Notes by William Lawrence, Esq. F. R. S. Surgeon to St. Bartholomew's Hospital, &c. &c. Second Edition, revised and augmented, by William Coulson, Demonstrator of Anatomy at the Medical School, Aldersgate-st. &c. &c. 8vo, pp. 379, 8 Plates. Simpkin and Marshall, London, 1827.

☞ *This Work is on a far smaller scale than the preceding, and not near so well adapted for the study of comparative anatomy. It will be much relished by those who have made advances in that useful and highly ornamental science.*

5. L'Agent immediat du Mouvement Vital devoilé dans sa Nature et dans son Mode d'Action, chez les Vegetaux, et chez les Animaux. Par M.H. Dutrochet, &c. &c. 8vo, pp. 226. Bailliere, Bedford Street, Bedford Square, 1827.

6. A concise Description of the Locality and Distribution of the Arteries in the Living Body. By G. D. Dermott, Lecturer on Anatomy and Surgery in the Anatomical School, Little Windmill Street. 12mo, pp. 144, with three Diagrams. Highley, London, 1827.

7. Memoirs of West Indian Fever; constituting brief Notices regarding the Treatment, Origin, and Nature of the Disease commonly called Yellow Fever. By John Wilson, M. D. R. N. 8vo, pp. 217. Burgess and Hill, London, 1827.

☞ *See our present number.*

8. A Treatise on those Diseases which are either directly or indirectly connected with Indigestion: comprising a Commentary on the principal Ailments of Children. By David Uwins, M. D. &c. 8vo, pp. 274. Underwoods, London, 1827.

9. The Lectures of Sir Astley Cooper, Bart. F. R. S. Surgeon to the King, &c. on the Principles and Practice of Surgery; with additional Notes and Cases. By Frederick Tyrrell, Esq. Surgeon to St. Thomas's Hospital, &c. &c. Vol. III. 8vo, pp. 538, two Plates. Simpkin and Marshall, London, 1827.

---

10. Two Letters, shewing the Impropriety of electing Assistant Surgeons. Respectfully addressed to the Governors of the Norfolk and Norwich Hospital. By John Cross, one of the Surgeons to the Hospital. 8vo, stitched, pp. 24. Norwich, 1827.

---

11. The Medico-Chirurgical Transactions, Vol. XIII. Part II. London, Longman and Co.

---

12. A Treatise on the Diseases of the Chest, and on Mediate Auscultation. By R. T. H. Laennec, M. D. &c. &c. Second Edition, greatly enlarged. Translated from the French, with Notes, and a Sketch of the Author's Life. By John Forbes, M.D. Member of the Royal College of Physicians, &c. 8vo, pp. 722, with Plates. Price 24 Shillings. Underwoods, November, 1827. ☞ *See Review.*

---

13. A Treatise on the Cutaneous Diseases incidental to Childhood; comprehending the Origin, Nature, Treatment, and Prevention. By Walter C. Dendy, Surgeon to the Royal Infirmary for Children, &c. &c. 8vo, pp. 289, with Plates. John Churchill, Leicester Square. London, November, 1827.

---

14. Medical Botany, up to No. XII. for Nov. 1, 1827. By Dr. Stephenson and Mr. Churchill.

☞ *See Review of this No.*

---

15. Anatomical Description of the Reflections of the Peritoneum and Pleuræ; with Diagrams. By G. D. Dermott, Lecturer on Anatomy and Surgery in the Anatomical School, Little Windmill-st. 8vo. stitched, pp. 24. Highley, London, 1827.

☞ *This little Work of Mr. Dermott's appears to us to be well adapted to the ends proposed. Different sections of the thorax and abdomen are given, in Lithographic Diagrams, which display the different inflections of their investing membranes in a clear and satisfactory manner. We can safely recommend the Work to the Anatomical Student.*

16. A Memoir on the Diagnostic Signs afforded by the Use of the Stethoscope, in Fractures, and in other Surgical Diseases. Translated from the French of Professor Lisfranc, with Notes and Additions, by J. R. Alcock. Pp. 36, with one Plate. Burgess, London, 1827.

☞ *We have looked over this translation, and have no hesitation in declaring that it is extremely well executed, whilst the notes and observations appended do credit to the industry and talent of the young author. The stethoscope, we have no doubt, may be applied to more purposes than were originally contemplated by the illustrious Laennec; but we really despair of its use in such cases as fracture, by our hospital and* private *optimists, when they doggedly scout its employment in diseases of the thorax.*

---

17. The Dissector, price 8d. Nos. 1 to 9.

☞ *We beg to return our best thanks to the Editors for their courtesy. We shall take care to supply them with our own Journal in exchange.*

---

18. A Letter addressed to the Faculty of Physic in London, on the Illegal and Unwholesome Bye-Laws of the College of Physicians of London, establishing a Monopoly in favour of the Graduates of Oxford and Cambridge. By H. Robertson, M. D. of the University of Edinburgh. 8vo. pp. 31, stitched. London, 1827.

☞ *The "Faculty of Physic" exists merely in Dr. Robertson's imagination. There is no such thing in London. It would be an illegal institution. But we do not deny the existence of unwholesome bye-laws, and of monopolies. The physicians of England and Scotland should join in a petition to the Legislature for redress —but not form any illegal institutions.*

---

19. Pathological and Practical Researches on Diseases of the Brain and the Spinal Chord. By John Abercrombie, M.D. Fellow of the Royal College of Physicians of Edinburgh, &c. 8vo. pp. 444. Edinburgh, 1827.

---

20. Remarks on Fish-poisons. By E. D. Allison, M.D. of Leith.

---

21. A Manual of Surgical Anatomy, containing a minute Description of the Parts concerned in Operative Surgery, with the Anatomical Effects of Accidents, and Instructions for the Performance of Operations. By H. M. Edwards, D.M.P. Translated, with Notes, by Wm. Coulson, Demonstrator of Anatomy at the Medical School, Aldersgate Street, &c. 12mo. pp. 427. London, 1828.

☞ *This is not meant to supersede the ordinary Works on Anatomy, as it treats only of those parts with which we have to do surgically, in accidents or operations. We are sure we need say little in commendation of any thing which proceeds from the pen of our able countryman, Dr. Edwards; but, from the hasty glance which we have been enabled to cast over the pages, we have no hesitation in bestowing upon it our meed of applause, such as it is. The translation appears to be respectably executed; but really, looking at the notes, one would imagine Mr. Coulson to be deeper read in the Lancet than any other modern Work. In his quotations from that Work, however, he cannot be accused of plagiarism.*

---

22. An Oration, introductory to the Study of the Healing Art; delivered by John Charles Litchfield, F.L.S. Surgeon, on Friday, September 28, 1827, in the School of Surgery, Sidmouth Street, Gray's-Inn Road. 8vo. stitched, pp. 44. London, 1827.

---

23. A Sketch of the History, Composition, and Medicinal Properties, of the Springs of Leamington Spa; to which is appended, an Outline of the Rules for Drinking the Waters, Bathing, Diet of the Patients, &c. By Charles Loudon, M.D. &c. 8vo. pp. 46. Leamington Spa, 1827.

☞ *Unlike those pitiful supplications for sympathy, wherewithal most of our Watering Places ever abound, it is characterised by the utter absence of egotism and selfish pretension. Distant practitioners will derive assistance from it, in forming directions for the use of those patients to whom they may judge a course of the Leamington Waters advantageous; and they who, from whatever motives, may find it necessary to make a temporary or permanent residence at this justly celebrated Spa, should take the "Sketch" itself as a perspicuous and faithful guide in the management of health, or the treatment of disease.*

24. Observations on the Properties and Effects of the Expressed Oil of the Seed of Croton Tiglium; together with the Botanical History, and a correct coloured Engraving of the Plant. By John Frost, of Emanuel College, Cambridge, &c. &c. 8vo. stitched, pp. 40, with one Plate. London, 1827.

---

25. A Description of Read's Patent Syringe, with Public and Professional Testimonials of its superior Utility. By John Read. London, price one shilling.

---

26. Arnott's Elements of Physics, &c. Second Edition, enlarged.

---

27. Formulary for the Preparation and Employment of several New Remedies. Translated from the Sixth Edition of the Formulaire of M. Majendie. By Joseph Houlton, F.L.S. 12s. 6d.

---

The following works have been received from M. Baillière, of Bedford-st. Bedford Square.

1. Belmas, D. Traité de la Gystotomie Sus Pubienne. Avec Planches, en 8vo. Paris, 1827. Prix 6s.
2. Desruelles, H. M. J. Traité de la Coqueluche, d'apres les Principes de la Médécine Physiologique. En 8vo. Paris, 1827. 6s.
3. Calmeil, (L. F.) de la Paralysie, considérée chez les Alienes Recherches, faites dans le Service de Feu, M. Royer Collard, et de M. Esquirol. En 8vo. prix 6s. 6d.
4. Ratier, Formulaire Pratique des Hôpitaux Civils de Paris, ou Recueil des Prescriptions Médicamenteuses employées par les Médécins et Chirurgiens de ces Etablissements. 3me Edition, revue et considerablement augmentée, 1 vol. en 18mo. 1827. 4s. 6d.
5. Gall, (I. I.) sur les Fonctions du Cerveau. 6 vol. en 8. Paris, 1826. 2l. 2s.
6. Roche, (L. Ch.) de la Nouvelle Doctrine Medicale, considerée sous le Rapport des Theories et de la Mortalité, Discution entre Messrs. Miquel, Bousquet, et Roche. Paris, 1827. En 8vo. 4s. 6d.
7. Tiedemann et Gmelin, Recherches Experimentales Physiologiques et Chimiques, sur le Digestion, considerée dans les quatre Classes d'Animeaux Vertébrés. Traduites de l'Allemand, par A. J. L. Jourdan, D. M. P. 2 vol. en 8vo. Paris, 1827. 15s.

8. Hoffbauer, (J. G.) Médécine Legale, relative aux Aliénés, et aux Sourds-Muets, ou les Lois appliquées aux Désordres de l'Intelligence. Traduite de l'Allemand, sur la derniere Edition, par A. M. Chambeyron, D.M.P. avec des Notes, par Messrs. Esquirol et Itard. En 8vo. Paris, 1827. Prix 7s.

8. Polinière, A. P. I. Etudes Cliniques sur les Emissions—Sanguines Artificials, Ouvrage qui a remporté le Prix proposé par la Sociétè Academique de Marseille, pour l'Anné 1826. 2 vol. en 8. Paris, 1827. Prix 12s.

9. Meckel (J.F.) Manuel d'Anatomie generale, descriptive, et pathologique. Traduit de l'Allemand, et augmenté des Faits Nouveaux dont la Sçience s'est enrichie jusqu'à ce Jour. Par A. J. L. Jourdan, M. D. P. et Breschet, Chef des Traveaux Anatomiques de la Faculté. 3 vol. en 8. Paris, 1827. Price, 1*l*. 5*s*.

10. Rayer, Traité Théorique et Pratique des Maladies de la Peau, fondé sur de Nouvelles Recherches d'Anatomie et de Physiologique Pathologique, avec 10 planches coloriées, 2 vol. en 8. Prix, 1*l*. 8*s*.

☞ *This is a very meritorious publication. The plates are well executed.*

J. B. Baillière, Foreign Bookseller, 3, Bedford Street, Bedford Square, London; Même Maison, Paris, Rue de l'Ecole de Medecine, No. 13.

### Miscellanies.

### ADDRESS

### *To the Subscribers of the Medico-Chirurgical Review.*

We earnestly solicit the attention of our readers to a subject which interests *them* at least as much as it does *ourselves*. In the first place, we beg to inform them that, ever since the commencement of our labours, it has been our uniform practice, to commit a new number to press on the very day that its predecessor had gone forth to the public. By this procedure, the editorial and typographical—or, in other words, the literary and mechanical, operations went on continuously and simultaneously through the quarter, at precisely the same rate, viz. three sheets, or 48 pages, every fortnight, so that neither the writers nor the printers had any relaxation at the beginning of the quarter, or any accumulation of labour towards its conclusion. This plan, which, we believe, is peculiar to ourselves, accorded with our habits of unremitting industry, and conduced much to order and punctuality at least, if not to many other useful ends. To it we owe much of our success—and from this plan we shall never deviate, so long as we continue journalists.

But we have had great reason to regret, that much of the benefit to which such a rigidly systematic, discipline might fairly lay claim, was denied to us, by the regulation which consigned our *printed sheets* to the warehouse, during a space varying from *one to thirteen weeks* after they were worked off! Thus, our analyses of books—our analytical selections from foreign Journals—our commentaries on the various productions of the periodical press at home—and our observations on the passing events of the medical world around us, were kept from the light, by the slow phases of a quarterly publication, till we saw them more or less anticipated by cotemporaries, who had no other power for such anticipations, but what the mere *mechanical celerity* of their vehicles afforded them—and who, in short, forestalled us—"*non vi sed sæpe cadendo.*" We were thus brought into the field of competition for priority, with one or both our hands tied behind our backs. Under such disadvantages, it is wonderful that we should have stood our ground—much more that we should have advanced! A period, however, has arrived, when a variety of circumstances has induced us to claim the indulgence of being put on something like equal terms with our competitors—a claim which a British public has never yet denied, and which, we are confident, the medical profession will not withhold from us. We ask no more—nay, we ask not quite so much. We desire only that the *mecha-*

*nical facility* of laying before the public the results of our labours in a *certain manner or mode*, which quadrates with our habits of industry; and then, if we are found wanting in the balance of merit, we shall consent to be struck off the list of candidates for public patronage.

In making this request, we impose no pecuniary tribute on our subscribers—we alter not the size, the arrangement, the construction, the objects, or the price of our journal. We only ask that those printed sheets which lie from one to thirteen weeks in the printing-office, may appear in fasciculi, or parts, at *half-monthly*, as well as at *trimestral* periods. To those who startle at innovation, we would put this plain question:—Can there be any objection, that each packet or fasciculus of three sheets, or 48 pages, of this Journal, should go forth to those who wish to have it every fifteen days, (half-monthly) instead of remaining in the printing-office for the space of many weeks? Each packet or fasciculus will contain a sixth part of the quarterly Number, with the exact proportion of expence affixed; and these fasciculi will bind up, at the end of the quarter, in precisely the same form, size, and price as they have hitherto done—the subscribers having the option of taking in the parts *half-monthly*, or the entire Number at the end of each quarter. The proposition is so reasonable, that we cannot anticipate any valid objection to it; and we are convinced that the great majority of our readers will highly approve of a measure which carries with it not a single disadvantage, but a host of facilities for the rapid diffusion of professional information.

The products of the periodical press have multiplied, and are daily multiplying, at such a rate, as to render our Periscope a department of at least as much importance as that of our Analytical Reviews. On this account, each Number of the Journal has lately been divided into two nearly equal portions—which division will apply to the fasciculi with as much convenience as to the integral Numbers, and without the slightest disruption of any of their constituent plans or principles. The two divisions of Review and Periscope will be so arranged, by folios and signatures, as to fall into their respective places, at the completion of each number and volume, so that no difference whatever will appear to those who prefer the Journal in its quarterly form.

The above modification of publication has been rendered imperative by the taste of the times we live in, and with which taste we must and ought to comply. The present is a very inquisitive age—and, as far as medicine is concerned, it is rather a turbulent æra. Unhappily, our profession is torn with dissensions, and split into parties, which are irritated against each other by their respective organs of the press. Whatever good may ultimately result from these terrible collisions, it will not be denied, that much obloquy will fall on the profession generally from the intemperance of the partizans. If ever there was a time, when an IMPARTIAL as well as independent journal was necessary for the peace and prosperity of medicine, this is the period. The term *independent* has lately indeed been usurped as signifying amenability to no law of honour, decorum, or justice—but the privilege of *abusing* all parties, except that of which the literary organ is the furious advocate. To this kind of *independence* we do not aspire. But we can fearlessly aver, that this Journal is the *organ of no party*. Our objects are the advancement of knowledge, the harmony, and consequently the respectability of medical practitioners, the reform of abuses, the protection of private character, the exposure of public delinquencies. The promotion of all these desirable objects will be much accelerated by the facilities which the *mode* of publication now contemplated will afford us; and so confident are we that the utility of this measure will appear unequivocally, from the moment it is put in execution, that we shall appeal at once to the experimentum crucis—the test of experience.*

In the mean time we have one very earnest request to make, namely, that our subscribers will give positive instructions to their own respective booksellers, to *immediately* order the parts or fasciculi (for the first and fifteenth of every month) from their London correspondents, in order that we may be early enabled to calculate on the number to be done up in *half-monthly parts*, and the number to be left for the *quarterly* form. The first part of No. XVI. will be published on the 15th January, 1828; but orders should be given for it by the country-booksellers, by the *very first correspondence* they have with the London houses. We shall consider the *early* attention of our subscribers to this point as a personal favour conferred on ourselves, which we will gratefully remember. More depends on this *punctuality* of our subscribers than they can possibly be aware of, and we do calculate on an indulgence in this respect, which imposes no inconvenience on *them*, and will confer an important favour on *us*.

We have only to add one remark, addressed to our cotemporaries. It is

---

* Among the many advantages which will result from this mode of publication in *half-monthly* fasciculi, one very important one will accrue to medical societies and book-clubs, now so numerous over the kingdom—namely, the facility of distributing these fasciculi among the members at short periods through the quarter, thus obviating the delay and inconvenience attending the quarterly distribution, and the hurried perusal of a work, each number of which, now equals a good sized octavo volume. This will be a most important advantage to societies, as well as to individuals. Men, in the bustle of private practice, will read a fasciculus, when the size of a quarterly number would deter them from immediate perusal. By this improved mode of circulation, therefore, we shall attain all the advantages of early information, and facility of future reference.

this—that we adopt the new modification of publication with no view or intention of trenching on the departments which they have struck out for themselves. We repeat it, that we shall alter not one tittle of the present arrangement or construction of this Journal, which, on the improved plan of half-monthly and quarterly distribution, we believe to be, of all others, the best calculated to diffuse *select useful* information through all classes of the profession. Our work will still continue a REVIEW of books and PERISCOPE of journals—and not a vehicle for the publication of original dissertations, elementary lectures, &c. for which there are many other periodicals. In bringing the products of our literary labours into the public market, at those periods which we deem most advantageous for our readers and ourselves, we wage no war on our contemporaries; while we *avowedly* compete with all and each of them in the useful occupation of collecting and concentrating the greatest quantity of practical matter into the smallest space. At the same time we will, most assuredly, direct all the power of legitimate criticism, which in us lies, (and that in a fair, open, and, we hope, fearless manner,) against error, misrepresentation, abuses, and *abuse;*—in short, against every thing, publicly printed, which we conceive to be contrary to sound principles of theory and practice, or subversive of that dignity, good faith, and liberality, which should characterise the productions of the medical press.*

We claim no exemption from criticism, and expect no indulgence for our errors. We will freely censure and freely commend—but we are determined not to be drawn into vulgar abuse on one hand, nor fulsome adulation of *partizans* on the other. In all our commentaries the support of TRUTH shall be our first object, and the detection of error our second. By our past and future labours the public will be enabled to judge how far we are competent to the task.

N.B. All articles EXTRA LIMITES, (that is, beyond the three sheets of which each Fasciculus will consist,) must be entirely at the private expence of the writers. We should prefer, indeed, that the authors would print those articles in their own way, and send them to our publisher, who will circulate them with the Journal.

---

* As we are enlisted under no banners but those of TRUTH and JUSTICE—we uphold not the sic volo sic jubeo institutions of overbearing medical aristocrasies—nor will we try to inflame the passions of wild and brawling democrats, who would set the whole profession in arms against each other, and thus lower them in the eyes of all enlightened people in this and other countries, solely for the purpose of puffing off a small faction, and filling the coffers of an individual.

### LIST
OF
### SUCCESSFUL CANDIDATES
FOR
### THE HOSPITAL REPORT PRIZE.

*Palmam qui meruit ferat.*

| NO. | NAME. | HOSPITAL. | DATE. |
|---|---|---|---|
| 1 | Mr. Thos. H. Smith, (Pupil) | St. Thomas's. | April, 1827. |
| 2 | Mr. George Bury, (Pupil.) | Winchester. | July, 1827. |
| 3 | Mr. T. Fereday, M.R.C.S. | St. Bartholomew's | Octr. 1827. |
| 4 | Mr. C. W. Turner, (Pupil.) | Cheltenham. | Octr. 1827. |
| 5 | Mr. G. Wickham. | Guy's. | Jany. 1828. |
| 6 | Mr. B. Eddison. | Nottingham. | |

### ADDITIONAL SUBSCRIBERS.

Brookes, Mr. Bedford-street, Covent Garden.
Clarke, H.P. Esq. Surgeon, 21st Fusileers, Rathdrum, Ireland.
Dewar, Dr. R. N.
Fereday, Mr. T. Member of the Royal College of Surgeons, Dudley.
Hugo, Mr. Surgeon, Crediton, Devon.
King, Mr. George, Surgeon-Apothecary, Northumberland-place, Bath.
Mac Andrew, Dr. to Calcutta.
Macansh, Mr. John, Surgeon.
Paget, Mr. John, Jun. Student in Dublin.
Peebles, Dr. Physician, Rome.
Podmore, Mr. Surgeon, Lewes, Kent.
Runciman, Mr. John, Surgeon, R.N.
Tweedie, Dr. Ely-place, Holborn.
Van Burm, Dr. John J. H. Trinidad.
Wallis, Mr. Surgeon, Dorchester.
Yates, Dr. Torrington-square.

